# Hybrid modeling of science and math teachers' psychological preparedness for AI- integrated teaching environment: EGA perspective of instructional proficiency, professional motivation, technophilia and needs of training

**DOI:** 10.3389/fpsyg.2026.1704162

**Published:** 2026-04-23

**Authors:** Usama M. Ibrahem, Tahani M. Alrosaa, Hanan M. Diab, Jawaher S. Alrashood, Hany M. Hagar, Sarah B. Alotaibi, Mahmoud A. Moussa

**Affiliations:** 1 Education College, University of Hail, Ha'il, Saudi Arabia; 2 Department of Educational Technology, College of Education, Suez Canal University, Ismailia, Egypt; 3 Department of Curricula and Teaching, College of Education and Human, Princess Nourah bint Abdulrahman University, Riyadh, Saudi Arabia; 4 Department of Computer Science, Applied College, University of Hail, Ha'il, Saudi Arabia; 5 College of Education and Human Development, Princess Nourah bint Abdulrahman University, Riyadh, Saudi Arabia; 6 Department of Sports Sciences and Physical Activity, College of Education, University of Hail, Ha'il, Saudi Arabia; 7 Suez Canal University, Ismailia, Egypt

**Keywords:** artificial intelligence (AI), EGA perspective, hybrid modeling, instructional proficiency, needs of training, professional motivation, psychological preparedness, technophilia

## Abstract

This study explored teachers' psychological readiness for integrating AI into teaching, focusing on cognitive, affective, and contextual factors. Using a 27-item Psychological Preparedness for AI-Integrated Teaching scale and single-item measures, science (*n* = 691) and mathematics (*n* = 231) educators were assessed. A multi-group confirmatory factor analysis (CFA) with WLSMV estimation confirmed factorial structure and invariance across disciplines, ensuring cross-group comparability. Exploratory Graph Analysis (EGA) with Walktrap community detection identified three dimensions: cognitive-affective readiness, technological-professional competence, and institutional support. Bootstrap analyses validated the stability of this structure. The findings offer a psychometrically sound, multidimensional framework for teacher readiness in AI integration, emphasizing individual and systemic influences on adaptive educational practices.

## 1 Introduction

The rapid integration of artificial intelligence (AI) into contemporary educational systems is fundamentally transforming teaching practices in science and mathematics education. AI-enabled technologies, including adaptive learning pathways, automated assessment, learning analytics, and intelligent instructional support, offer promising opportunities to improve educational quality and effectiveness. However, the successful implementation of these technologies depends critically on teachers' psychological readiness to engage with AI-integrated teaching environments (Makhmudova et al., 2025; Kyrpa et al., 2024).

A growing body of research indicates that teachers‘ readiness to integrate AI is a multidimensional psychological construct, encompassing cognitive, affective, and contextual factors. Effective engagement with AI requires a combination of pedagogical competence, sustained professional motivation, positive technological attitudes, and access to appropriate training opportunities (Shi and Zhang, 2025; Aghaziarati et al., 2023). Despite this recognition, existing studies have largely addressed these factors in isolation, providing limited insight into how they collectively and interactively shape teachers' readiness for AI-integrated instruction.

This gap is particularly evident in science and mathematics education, where AI applications demand higher levels of instructional decision-making, data interpretation, and pedagogical adaptation (Makhmudova et al., 2025; Yang and Wang, 2024). Traditional linear models and regression-based approaches are inadequate for capturing the dynamic, interconnected nature of teachers‘ psychological readiness. To address this limitation, the present study adopts a hybrid modeling approach based on Exploratory Graph Analysis (EGA), which enables the identification of underlying psychological dimensions and the investigation of their network structure. Specifically, this study examines how instructional proficiency, professional motivation, technophilia, and perceived training needs interact to shape science and mathematics teachers' psychological preparedness for AI-integrated teaching environments.

## 2 The problem

The rapid integration of AI into educational systems is causing a radical transformation in teaching and learning processes, especially in science and mathematics education. AI-integrated teaching environments include intelligent guidance systems, adaptive learning platforms, automated assessment tools, and data-driven educational support, imposing new cognitive, professional, and psychological requirements on teachers (Mandal, 2024). While these developments offer tremendous educational potential, the psychological readiness of teachers to effectively interact with AI-integrated teaching environments remains variable and not well understood.

Recent evidence indicates that teachers‘ readiness to integrate AI goes beyond technical proficiency and is influenced by a range of psychological and motivational factors, including teaching competence, professional motivation, attitudes toward technology, and the adequacy of received training (Silagan, 2025; Shi and Zhang, 2025). Some science and math teachers exhibit high levels of enthusiasm, confidence, and adaptability when using AI-supported education, while others face uncertainty, resistance, or hesitation toward AI-driven educational change. These disparities highlight the importance of studying not only teachers' skills but also their deep psychological attitudes toward the use of AI in educational contexts (Aghaziarati et al., 2023; Bakracheva and Mizova, 2024).

The skills gap may result in the underutilization of AI's potential and impede its effective incorporation into science curricula (Kohnke et al., 2025). A significant challenge is the possibility for heightened workload and time commitment for teachers. Implementing AI tools requires time for lesson design, modifying current resources, and acquiring proficiency in new software (Javed, 2024; Njuguna, 2024). Teachers may also need to dedicate time to resolving technical issues and offering personalized assistance to students utilizing AI-driven learning platforms (Kohnke et al., 2025; Xie et al., 2017).

Despite the increasing studies on AI in education, current research has largely focused on technological capabilities, student learning outcomes, or teachers‘ general attitudes toward digital tools. Much less attention has been given to how multiple psychological dimensions interact simultaneously to shape teachers' readiness for AI-integrated teaching environments. There is a notable lack of comprehensive models that study the interrelationships between teaching competence, professional motivation, technology inclination, and perceived training needs, considering them as interconnected components of teachers' psychological readiness (Dahri et al., 2024; Aghaziarati et al., 2023). Most previous studies have addressed these concepts in isolation from each other, relying on linear or regression approaches that do not cover the complexity of teachers' real experiences in AI-rich educational environments (Mandal, 2024; Shi and Zhang, 2025).

This gap is particularly evident in the field of science and math education, where AI applications require higher levels of educational decision-making, data interpretation, and pedagogical adaptation (Makhmudova et al., 2025). Teachers in these disciplines are expected not only to operate AI tools but also to effectively integrate them into curriculum design, classroom interaction, and assessment practices (Kyrpa et al., 2024). As a result, the psychological readiness for AI-integrated teaching involves a delicate balance between competence, motivation, openness to technology, and access to targeted professional training (Makhmudova et al., 2025; Low et al., 2025).

Traditional linear modeling approaches struggle to explain these complex relationships. Researchers tend to assume the existence of unidirectional relationships, ignoring the possibility that psychological factors may simultaneously act as drivers, mediators, or constraints within a broader system. To address this shortcoming, this study adopts a hybrid modeling methodology, integrating Exploratory Graph Analysis (EGA) within a network psychometrics framework. This approach allows for the identification of underlying psychological dimensions, mapping their interconnections, and uncovering central and influential factors within teacher readiness networks.

By applying this methodology, the study aims to clarify how the aggregation and interaction of teaching competence, professional motivation, passion for technology, and training needs contribute to the psychological readiness of science and mathematics teachers for AI-integrated teaching environments. Understanding these interactions is crucial for designing targeted professional development initiatives that surpass conventional training models and instead address the specific psychological factors that facilitate or hinder the adoption of AI.

In response to international calls to enhance teachers‘ capabilities in line with digital transformation and sustainable education programs (Vičič Krabonja et al., 2024; Butler et al., 2024), this study aims to bridge a critical research gap by providing a network-based understanding of teachers' psychological readiness. Thus, the study contributes both theoretically and practically to the ongoing dialogue on integrating AI into science and mathematics education, providing evidence-based insights to enrich policies, teacher training, and institutional support strategies.

## 3 Questions

What is the underlying factor structure of the Psychological Preparedness for AI-Instructional Integration instrument among teachers?

Is the factor structure of teachers' psychological preparedness invariant across academic disciplines (Science vs. Mathematics)?

Does Exploratory Graph Analysis reveal distinct, conceptually meaningful dimensions of the study for AI integration in the teaching environment?

## 4 Theoretical Framework

### 4.1 The Impact of Teachers' Psychological Readiness on the Learning Environment

Teachers' psychological readiness plays a pivotal role in shaping the quality and effectiveness of AI-integrated teaching environments, particularly in science and mathematics education. Psychological factors, such as attitudes toward technology, teaching self-efficacy, and levels of liking or aversion to technology, significantly influence teachers' instructional choices and classroom practices (Nordlöf et al., 2019). Teachers with high psychological readiness tend to experiment with AI-enabled tools, adopt innovative teaching strategies, and design adaptive learning experiences that respond to diverse student needs (Dursun, 2019). Conversely, teachers who are unprepared or apprehensive about AI tend to rely on traditional, teacher-centered approaches, thus limiting the pedagogical potential of AI to improve student learning outcomes (Stumbriene et al., 2024; Joo et al., 2018).

Empirical evidence suggests that teachers' confidence and enthusiasm for using AI directly impact student engagement and motivation. When teachers demonstrate positive attitudes and proficiency in using AI-enabled tools, students are more likely to show curiosity, maintain focus, and actively participate in learning activities ((Dursun, 2019; Yang and Wu, 2024). AI-enabled teaching systems, including adaptive feedback mechanisms and personalized learning pathways, also enhance student engagement by accommodating individual learning paths and fostering collaborative learning opportunities ((Nordlöf et al., 2019). Thus, teachers' psychological readiness extends beyond individual practice to influence the broader social and emotional climate of the classroom.

At the classroom level, psychologically prepared teachers contribute to the development of an open and progressive learning culture characterized by experimentation, innovation, and critical inquiry. Teachers receptive to AI integration create environments that encourage students to explore complex problems, engage in higher-order thinking, and develop the future competencies necessary for an AI-driven society (Butler et al., 2024). Conversely, resistance to AI adoption may lead to less flexible and inclusive learning environments, and less support for student-centered teaching methods (Butler et al., 2024).

Furthermore, teachers' psychological readiness has significant implications for equity and equal opportunity in AI-integrated classrooms. Confident and well-prepared teachers are better positioned to ensure that all students, regardless of their backgrounds or learning differences, benefit from AI-enhanced learning. This includes providing equal access to AI resources, adapting instruction to diverse learning styles, and carefully monitoring potential algorithmic biases inherent in AI systems (Jacobson, 2025; Joo et al., 2018).

Therefore, enhancing teachers' psychological readiness requires a holistic approach that addresses attitudes, self-efficacy, motivation, and concerns related to AI use. Research underscores the importance of targeted professional development, ongoing mentoring, and institutional support structures that foster trust, ethical awareness, and shared responsibility in AI adoption (Jacobson, 2025; George and Wooden, 2023). These combined efforts enable schools to create innovative, engaging, and inclusive learning environments, thereby preparing science and mathematics education to effectively meet the demands of an AI-integrated learning environment.

### 4.2 Learning theories as a framework for the effectiveness of AI-supported teaching

The integration of AI into educational environments has profound pedagogical implications, particularly for science and mathematics teachers working in AI-integrated learning environments. Understanding how AI contributes to improved teacher effectiveness requires connecting this transformation to learning theories that explain how knowledge is constructed, transferred, and organized (Atabekova et al., 2025; Orsoni, 2024). These theoretical perspectives provide a coherent framework for meaningfully explaining teachers' psychological readiness—including teaching competence, professional motivation, technological aptitude, and training needs.

Constructivist learning theory, as formulated by Piaget and Vygotsky, asserts that learning is an active, socially connected process in which learners construct knowledge through interaction with their environment. In AI-integrated learning environments, this perspective redefines the teacher's role from a transmitter of knowledge to a facilitator of learning (Low et al., 2025). AI applications, such as intelligent teaching systems, adaptive learning platforms, and real-time learning analytics, activate constructivist principles by providing personalized feedback, facilitating complex tasks, and supporting differentiated instruction based on learner needs (Lajoie and Li, 2023; Vorsah and Oppong, 2024). For science and mathematics teachers, these capabilities alleviate the burden of routine teaching tasks, such as frequent assessment and error detection, allowing for a greater focus on high-level instructional design and conceptual guidance, thereby enhancing teaching efficiency and perceived teaching effectiveness.

From a social learning perspective, Bandura's theory emphasizes the importance of observation, modeling, and interaction in learning processes. AI-powered collaboration platforms, virtual labs, and simulation environments expand opportunities for social interaction and shared meaning in science education (Strielkowski et al., 2025). These tools enable teachers to model expert reasoning, guide collaborative inquiry, and monitor group dynamics more effectively than in traditional classroom settings (Lajoie and Li, 2023; Gkintoni et al., 2025). AI-enhanced formative assessment supports teachers by providing real-time insights into learner interaction and collaboration patterns, thereby enhancing classroom management and pedagogical responsiveness (Atabekova et al., 2025). These capabilities positively contribute to teacher motivation and confidence in adopting AI-supported teaching practices.

Cognitive load theory (CLT) offers further explanation by focusing on the mental effort required for learning. Teaching science and mathematics often involves abstract concepts and solving complex problems, making cognitive load management crucial in the educational process. AI systems support cognitive load theory by segmenting content, providing visual representations, and adaptively sequencing learning tasks to reduce cognitive overload (Lajoie and Li, 2023; Gkintoni et al., 2025). For teachers, AI-powered analytics automate progress monitoring and provide evidence-based instructional recommendations, enhancing decision-making accuracy and teaching efficiency (George and Wooden, 2023; Atabekova et al., 2025).

The concept of interconnected learning portrays learning as the formation of networks across digital tools, information sources, and social systems. AI-integrated teaching environments embody this model by distributing learning among human and non-human elements. In this context, teacher effectiveness depends not only on their content expertise but also on their ability to guide, organize, and sustain learning networks (Gkintoni et al., 2025). AI supports this process by aggregating information, recommending resources, and creating personalized learning content, thereby fostering adaptability and lifelong professional learning (Wang et al., 2023; Sajja et al., 2024; Correia et al., 2024).

These theoretical perspectives collectively affirm that AI enhances teacher effectiveness not only through technological enhancement but also through its alignment with fundamental learning theories that shape teachers' psychological readiness for AI-integrated teaching environments.

**Figure 1:**
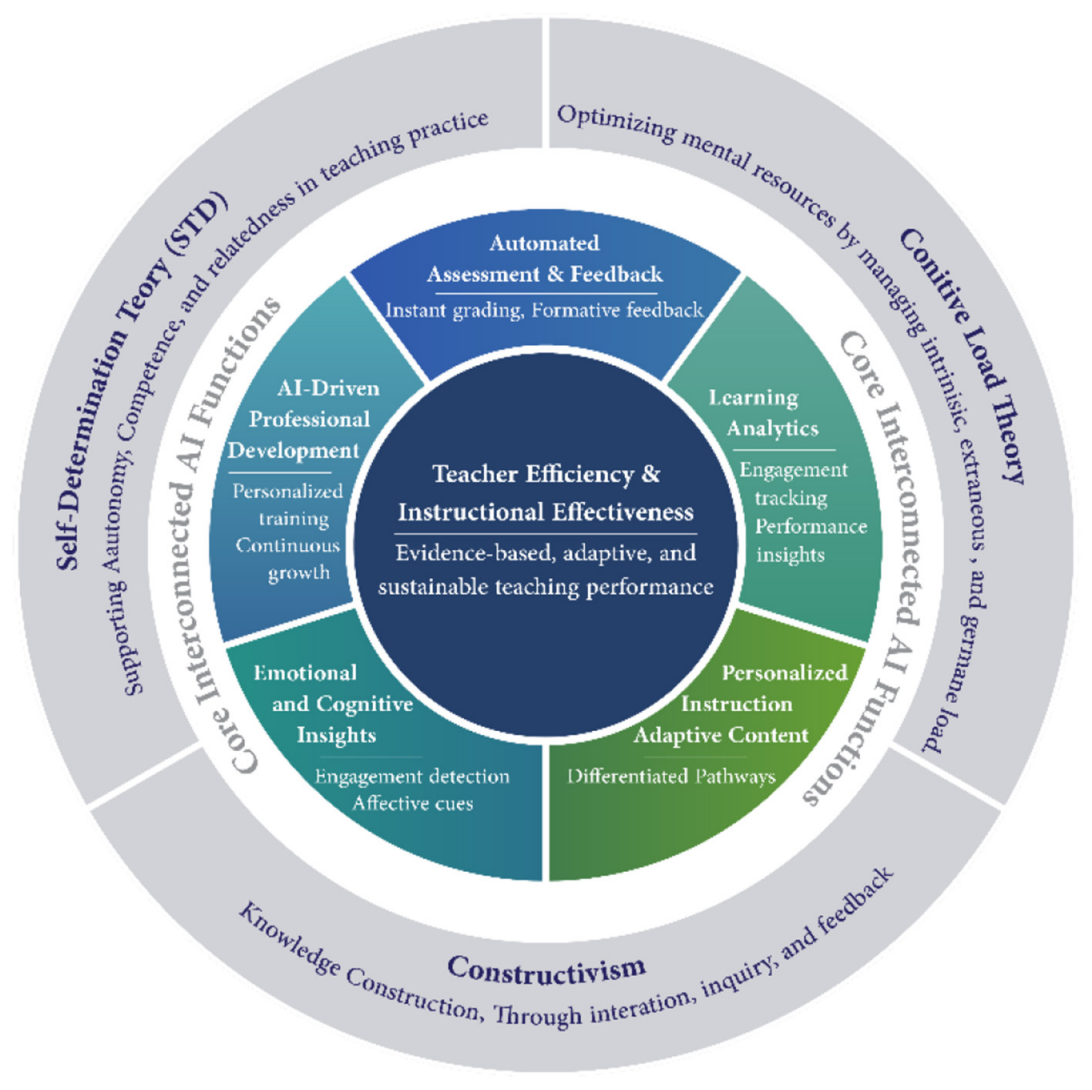
Enhancing teacher effectiveness using AI based on learning theories.

This Figure 1 illustrates a theoretical framework demonstrating how constructivist AI systems, cognitive load theory, and self-determination theory can enhance teacher effectiveness through automated assessment, learning analytics, personalized instruction, emotional understanding, and AI-supported professional development.

### 4.3 Teachers' Pedagogical Competence and Professional Motivation in Their Interaction with AI

Teachers' pedagogical competence and professional motivation are key factors in determining how effectively AI is integrated into science and mathematics teaching. To effectively utilize AI, teachers need more than just technical knowledge; they must be able to make pedagogical decisions that help them connect AI tools to learning objectives, teaching methods, and their students' cognitive needs. Teachers with high pedagogical competence are better positioned to critically evaluate AI tools, employ them to simplify complex concepts, and use data-driven insights to improve instructional decision-making (Simut et al., 2024; Wang, 2025).

In AI-integrated teaching environments, pedagogical competence increasingly encompasses adaptive instructional design, interpreting learning analytics, and coordinating human-AI collaboration in the classroom (Acosta-Enriquez et al., 2025). Advanced pedagogical competence enables teachers to leverage AI to personalize learning, provide timely feedback, and support differentiated instruction (Vistorte et al., 2024). Conversely, limited pedagogical competence may lead to superficial or fragmented use of AI, adding little pedagogical value.

Professional motivation also influences teachers' engagement with AI by affecting their openness to innovation, their perseverance in learning new practices, and their commitment to professional development (Acosta-Enriquez et al., 2025). Teachers who are self-motivated and understand the importance of AI to their educational goals are more likely to experiment with AI-enabled strategies and continue using them long-term (Howard et al., 2025). In contrast, low motivation or externally imposed adoption may lead to resistance or limited engagement (Sun et al., 2023).

Importantly, pedagogical competence and professional motivation interact dynamically. Motivated teachers are more likely to seek training that enhances their AI-related pedagogical skills, while increased competence boosts motivation by fostering self-efficacy and professional autonomy (Acosta-Enriquez et al., 2025). From a network perspective, these concepts act as interconnected and influential elements within teachers' psychological readiness, shaping related tendencies such as a love of technology and perceived training needs (Sun et al., 2023; Low et al., 2025).

Recognizing this interaction is essential for designing professional development initiatives that support the sustainable and pedagogically grounded integration of AI in science and mathematics education.

### 4.4 Technophilia as a dispositional factor shaping teachers' responses to AI innovation

Technophilia refers to a positive psychological inclination toward technology, characterized by curiosity, enjoyment, and openness to using emerging digital tools. In the field of science and mathematics education, a love for technology is a major personal factor that influences teachers‘ psychological readiness for AI-integrated teaching environments. With the increasing role of AI in curriculum design, assessment, and classroom interaction, teachers' technological inclinations determine whether AI is viewed as a supportive educational resource or a disruptive challenge (Hu et al., 2025).

Recent research indicates that teachers with higher levels of technological affinity are more inclined to explore AI-based educational applications, experiment with innovative teaching strategies, and persist in learning new technologies despite initial difficulties (Howard et al., 2025; Bakracheva and Mizova, 2024). These teachers tend to approach AI with an adaptive and exploratory mindset, viewing technological change as an opportunity for professional development rather than a threat to established educational practices. This approach supports the integration of AI more effectively and sustainably in the teaching of science and mathematics.

Conversely, a low inclination toward technology is often associated with hesitation, avoidance, or reliance on traditional teaching methods, which limits the educational potential of AI-supported environments (Hu et al., 2025; van der Klink and Alexandrou, 2022). It is important to emphasize that the inclination toward technology does not mean accepting it uncritically, but rather reflects a desire to engage consciously and thoughtfully with innovation while maintaining sound educational judgement. This distinction is particularly highlighted in science and mathematics classes, where educational rigor and conceptual coherence remain essential.

From a psychological readiness perspective, the inclination toward technology interacts dynamically with teaching competence, professional motivation, and perceived training needs (Simut et al., 2024; Wang, 2025). Teachers with a strong inclination toward technology are more likely to pursue targeted professional development, build competence through practice, and maintain motivation during the application of AI. Network-based analyses suggest that the inclination toward technology may play a pivotal or linking role in teacher readiness profiles, connecting motivational and competency dimensions (Acosta-Enriquez et al., 2025; Sun et al., 2023).

Perceiving the inclination toward technology as a personal factor has significant implications for teacher development. Professional development initiatives that cultivate positive technological attitudes alongside pedagogical skills can enhance teachers' responsiveness to adapting to AI innovations and support more effective learning environments integrated with AI (Hu et al., 2025; Javed, 2024).

### 4.5 Learning and professional development needs for integrating AI: supporting teachers in smart classrooms

The effective integration of AI in science and mathematics education heavily relies on providing purposeful learning and professional development opportunities for teachers. With the increasing use of AI tools in smart classrooms to personalize content, assessment, and educational support, teachers are required to develop not only technical knowledge but also pedagogical competence and psychological readiness to use these tools purposefully (Hu et al., 2025). Therefore, professional development plays a pivotal role in preparing teachers for AI-integrated teaching environments.

Recent studies confirm that traditional tool-oriented training is insufficient to support the sustainable integration of AI (Acosta-Enriquez et al., 2025; Stumbriene et al., 2024). Instead, teachers need professional learning experiences that combine knowledge of AI, educational application, ethical awareness, and reflective practice (Hu et al., 2025; Howard et al., 2025; Javed, 2024). In science and mathematics classrooms, where AI systems often mediate complex cognitive tasks, professional development should address how AI supports conceptual understanding, inquiry-based learning, and data-driven teaching. These learning opportunities enhance teachers' instructional efficiency and reduce the uncertainty associated with adopting AI.

Professional development needs are closely linked to teachers' motivations and technological inclinations. Teachers who see training as relevant to their teaching goals and the reality of their classrooms are more likely to engage effectively and persistently in developing AI-related competencies (Hu et al., 2025). Conversely, limited access to organized training or continuous support may enhance fears, technology anxiety, or resistance to AI-based innovation. Studies have shown that mentoring, peer collaboration, and school learning communities contribute to supporting teachers in practically applying training, enhancing confidence, and fostering professional learning in smart classroom contexts (Stumbriene et al., 2024).

It is important for professional development for integrating AI to be adaptive and continuous, not intermittent. Smart classrooms are evolving rapidly, requiring teachers to continuously update their knowledge and skills (Simut et al., 2024; Wang, 2025). Network theories on teacher readiness indicate that training needs interact dynamically with teaching competence, motivation, and passion for technology, shaping the overall profile of teacher readiness (Bakracheva and Mizova, 2024). Understanding these interconnections enables the design of more targeted and responsive professional development programs.

By aligning professional development with the psychological and educational needs of teachers, educational systems can better support science and mathematics teachers in utilizing AI as a valuable educational resource. This support is essential for developing effective, equitable, and future-ready teaching practices in AI-integrated learning environments.

### 4.6 Exploratory graphical analysis

Exploratory Graphical Analysis (EGA) provides a robust framework for investigating the structure and dynamics of psychological constructs at both dimensional and item levels. When standardizing instruments with multiple items nested within specific psychological domains, analyses typically proceed across two complementary levels: the dimensional (latent/composite) level and the item-level (network) level (Borsboom, 2008; Schmittmann et al., 2013). High-Resolution Networks (HRNs) represent an item-level analytic strategy, modeling each item as an independent node that captures fine-grained behavioral or psychological manifestations of a construct, rather than relying solely on total or dimension scores (Borsboom and Cramer, 2013; Epskamp et al., 2017; Fried and Robinaugh, 2020).

Within HRNs, edges represent partial associations between items, allowing precise examination of inter-item relationships and the identification of central or bridge nodes linking distinct psychological domains (Cramer et al., 2012; Fried et al., 2017; Jones et al., 2021). This framework enhances sensitivity for detecting emergent structures and dynamic interactions that may be obscured in traditional latent-variable analyses (Cramer et al., 2012; Robinaugh et al., 2020). Importantly, HRNs conceptualize psychological constructs as arising from interactions among items, rather than as latent sources generating responses. This perspective emphasizes process-oriented analysis, enabling the exploration of relational mechanisms, network influence, and potential targets for intervention (Haslbeck and Waldorp, 2018; Borsboom, 2022). Demographic or contextual variables can also be integrated into HRNs as predictors or moderators, facilitating a nuanced understanding of psychological dynamics (Haslbeck and Ryan, 2022).

While HRNs focus on item-level dynamics, EGA complements this approach by uncovering latent factor structures, determining dimensionality, and clustering items according to their emergent communities (Golino and Demetriou, 2017; Golino and Epskamp, 2017). Together, HRNs and EGA provide a multiscale perspective: EGA identifies coherent dimensions of psychological constructs, whereas HRNs quantify inter-item relationships, centrality, and bridging patterns within and between these dimensions (Bringmann and Eronen, 2018; Robinaugh et al., 2020).

In contrast, Low-Resolution Networks (LRNs) operate at the level of aggregated dimensions, examining interrelations among constructs rather than individual items (Epskamp et al., 2017). LRNs are particularly suitable for large instruments where item-level modeling may introduce noise, or when the primary focus is on dimensional interactions rather than internal item dynamics (Fried et al., 2017; Bringmann et al., 2019). Unlike HRNs, LRNs do not address factor structure or centrality but provide a macro-level understanding of how psychological dimensions influence one another (Borsboom and Cramer, 2013; Marsman et al., 2019).

## 5 Method

### 5.1 Data collection and procedure

The research protocol received ethical clearance from the College of Education Ethics Committee at Suez Canal University. Online informed consent was secured from all participants (Approval No. 4, approved on 24 November 2025).

Participation in the study was entirely voluntary. Prior to accessing the online questionnaire, participants were provided with detailed information about the purpose of the study, the nature of the survey, and their rights as participants. Electronic informed consent was obtained before participation. Participants were informed that they could decline to participate or withdraw from the survey at any time without any negative consequences.

To ensure confidentiality and anonymity, no personally identifiable information was collected. Data were gathered using a secure online survey platform and stored in password-protected files accessible only to the research team. All results were analyzed and reported in aggregated form to prevent individual identification.

The final dataset was complete, with no missing responses, enabling robust statistical analysis. This included Exploratory Graph Analysis (EGA) to model the interrelations among professional motivation, instructional proficiency, technophilia, perceived training needs, and psychological preparedness. The approach allowed for a network-based understanding of how individual, professional, and contextual factors collectively shape teachers' readiness to adopt AI-integrated teaching practices.

### 5.2 Participants

The study sample consisted of 922 teachers, including 691 science teachers and the remaining 231 mathematics teachers, recruited from various educational institutions. The participants were predominantly female (92.1%), with male teachers representing 7.9% of the sample. Participants' professional experience varied widely, with 51.8% reporting more than 15 years of experience, 27.8% between 10 and 15 years, 11.7% with less than 5 years, and 8.7% between 5 and 10 years.

Teachers' age distribution indicated that the majority were in the 41–50 year range (52.7%), followed by 31–40 years (29.1%), 51 years and above (12.7%), and 20–30 years (5.5%). Regarding educational qualifications, most participants held a bachelor's degree (74.6%), while 13.6% had a master's degree, 7.4% held postgraduate diplomas, and 4.4% possessed a doctoral degree.

In terms of institutional affiliation, the majority worked in public schools (89.7%), with smaller proportions employed in private schools (9.8%), Azhar schools (0.4%), or international schools (0.1%). The professional roles of participants were primarily teachers (93.2%), with smaller numbers serving as educational supervisors (4.0%), assistant teachers (1.5%), or school principals (1.3%). Participants were distributed across different educational stages, including the primary level (53.4%), middle school (25.4%), secondary school (16.3%), special education (1.8%), and kindergarten (3.1%).

Teachers' frequency of technology use in the classroom varied, with 74.1% using technology daily, 19.0% weekly, 6.9% monthly, and a small proportion reporting rare usage (2.7%). These demographic characteristics indicate a sample with substantial teaching experience, strong female representation, and high engagement with technology, providing a representative foundation for examining teachers' psychological readiness for AI-integrated instruction.

### 5.3 Hypotheses

Teachers' psychological readiness for AI-integrated teaching environments exhibits a multidimensional network structure.

Teaching competence and professional motivation will emerge as central nodes within the readiness network.

Technophilia and perceived training needs will show statistically significant correlations with teaching competence within the network structure.

### 5.4 Tools

Teachers' psychological preparedness for AI-integrated instruction was assessed using a structured questionnaire designed to capture their readiness in educational settings. Central to this assessment was a 27-item with 5 Likert-scale points Psychological Preparedness scale, grounded in a general factor model, which evaluated cognitive, affective, and contextual aspects of teachers' engagement with AI-driven instructional practices. This scale measured self-efficacy, confidence, and adaptive capacity, providing a comprehensive representation of teachers' ability to incorporate AI tools effectively into their pedagogy.

The 27-item Psychological Preparedness for AI-Instructional Integration scale was subjected to Confirmatory Factor Analysis (CFA) to rigorously evaluate its structural validity as a multidimensional measure of teachers' readiness.

In parallel, five single-item measures, each employing a 10-point response scale, were used to capture complementary professional and technological dimensions. Professional Motivation assessed the intrinsic drive and commitment of teachers to adopt AI-enhanced teaching strategies, while Instructional Proficiency reflected their perceived competence in designing and implementing AI-supported learning experiences. Technophilia evaluated teachers' positive orientation toward digital tools, encompassing curiosity, enthusiasm, and openness to technological innovation. Training Need measured perceived gaps in professional development, indicating areas where additional support might enhance AI adoption. Finally, Technology Satisfaction gauged teachers' contentment with the availability and effectiveness of existing technological resources. Collectively, this combination of multi-item and single-item scales provided a nuanced and robust measurement framework, enabling detailed exploration of the interrelations among professional motivation, pedagogical competence, technological disposition, and psychological readiness. These data were subsequently analyzed using Exploratory Graph Analysis (EGA) to model the networked structure of readiness, offering insights into how individual, professional, and contextual factors coalesce to influence teachers' adoption of AI in classroom instruction.

### 5.5 Statistical analysis

This manuscript deliberately employs two related yet conceptually distinct terms, 'Teaching Competence' and 'Instructional Proficiency', to reflect the necessary distinction between theoretical conceptualization and empirical operationalization within the study framework. In the theoretical sections (Sections 4.3, 4.4, and throughout the literature review), the term ‘Teaching Competence' is used to denote the broad, multidimensional construct encompassing educators' pedagogical knowledge, adaptive expertise, classroom management skills, and capacity to design effective learning experiences. This usage aligns with established educational literature, where competence is understood as a holistic integration of knowledge, skills, and dispositions that enable effective teaching practice (Simut et al., 2024; Wang, 2025; Acosta-Enriquez et al., 2025).

In contrast, within the empirical and methodological sections, specifically in the measurement tools, descriptive statistics (Table 2), and Exploratory Graph Analysis (EGA) model, the term ‘Instructional Proficiency' is employed to refer precisely to the operationalized, single-item measure of teachers‘ perceived ability to integrate AI into their instructional practices. This distinction is methodologically intentional and theoretically grounded: while the broader concept of “competence” informs the study's theoretical framework and hypothesis development, the empirical model requires a precisely defined, measurable node that captures the specific variance associated with teachers' self-perceived instructional capability in AI contexts.

This dual-terminology approach serves three critical purposes. First, it maintains fidelity to the theoretical literature, where ‘competence' is appropriately discussed as a multifaceted construct. Second, it ensures analytical precision within the network model, where ‘Proficiency' functions as a discrete, reliably measured node. Third, it transparently acknowledges the inherent gap between broad theoretical constructs and their empirical indicators, a gap that all quantitative research must navigate. By distinguishing between ‘competence' as a theoretical concept and ‘Proficiency' as an empirical measure, the study preserves theoretical richness while upholding methodological rigor, thereby enhancing both the interpretability and replicability of the findings.

All statistical analyses were conducted in R GUI 4.5, utilizing a combination of confirmatory, reliability, and network modeling frameworks. Preliminary data screening included assessment of item distributions, skewness, and kurtosis. Weak-loading items were identified and removed, and parcels were constructed to optimize model estimation. Confirmatory factor analysis (CFA) was performed using the lavaan package with the WLSMV estimator to account for the ordinal nature of the items. Multi-group CFA was employed to evaluate factorial invariance across groups, sequentially testing configural, metric, and scalar models. Internal consistency was assessed using McDonald's omega coefficients. Exploratory Graph Analysis (EGA) was conducted using the EGAnet package, applying the Glasso regularization algorithm and Walktrap community detection to estimate network structures among latent constructs. Bootstrap procedures were implemented to examine the stability and reproducibility of the network solution across 500 resamples, ensuring robust and replicable estimation.

This study employed a hybrid modeling approach within the Exploratory Graph Analysis (EGA) framework to comprehensively capture the multifaceted structure of teachers' psychological preparedness. The term “hybrid” here denotes the integration of two distinct types of nodes within a single psychological network: (a) latent dimensions represented by parcels derived from multi-item scales, and (b) standalone single-item measures. Specifically, the core construct of *Psychological Preparedness*, originally assessed via a 27-item scale, was operationalized using five empirically validated parcels (F1–F5) derived from confirmatory factor analysis. These parcels function as nodes that reflect the stable, shared variance of the latent cognitive-affective preparedness dimension. Simultaneously, five distinct professional and dispositional factors, *Professional Motivation, Instructional Proficiency, Technophilia, Training Need*, and *Technology Satisfaction*, were entered as individual nodes using their respective single-item measures. By combining parceled latent constructs with single-item indicators, this hybrid configuration leverages the psychometric reliability of aggregated dimensions while preserving the unique variance and specificity of discrete psychological attributes. Consequently, the network model captures both the broad, stable aspects of readiness and the specific, actionable teacher characteristics, enabling a more nuanced and ecologically valid understanding of the interrelationships shaping AI integration readiness in educational contexts.

To test Hypothesis 3, which proposed bivariate relationships among the single-item measures (Professional Motivation, Instructional Proficiency, Technophilia, Training Need, and Technology Satisfaction) and the parceled Preparedness construct, Pearson product-moment correlation coefficients were computed. Although single-item measures have been subject to historical concerns regarding reliability, contemporary methodological research has demonstrated that single-item measures are appropriate when the construct is narrowly defined, unidimensional, and readily accessible to respondents (Allen et al., 2022; Matthews et al., 2022). In the present study, each single-item measure was designed to capture a specific, concrete attribute (e.g., “My overall level of enthusiasm for using new technologies” for Technophilia) rather than a broad, multifaceted construct. Furthermore, single-item measures offer advantages in large-scale survey research, including reduced respondent burden and increased data quality (Fuchs and Diamantopoulos, 2009). The validity of these single-item measures was supported by their theoretically consistent relationships with the multi-item Preparedness scale in the subsequent network analysis, providing evidence of convergent validity. All correlation analyses were conducted using Pearson's r with listwise deletion, as the dataset contained no missing responses.

### 5.6 Results

#### 5.6.1 Psychological preparedness for AI-instructional integration structure and invariance across groups

A multi-group confirmatory factor analysis (CFA) was conducted to evaluate the factor structure of the 27-item instrument across science (*n* = 691) and Mathematics (*n* = 231) groups. Following preliminary analyses, three weak-loading items (Q8, Q13, Q16) were removed, and the optimized model comprised five parcels: F1 (Q1–Q3), F2 (Q4–Q6, Q14), F3 (Q7, Q9), F4 (Q10–Q12, Q15), and F5 (Q17–Q27), with F5 represented using two parcels to improve estimation. Models were estimated using WLSMV to account for the ordinal nature of items. Overall model fit was acceptable to good, with CFI = 0.992 and 0.987, TLI = 0.989 and 0.983, SRMR = 0.077 and 0.093 for Science and Mathematics groups, respectively; RMSEA values were elevated (0.116 and 0.127), likely reflecting model complexity and parceling. Standardized factor loadings were strong across both groups, ranging from 0.849 to 0.963 for parcels F1, 0.711 to 0.912 for F2, 0.847 to 0.899 for F3, 0.836 to 0.880 for F4, and 0.950 to 0.980 for F5 in Science, with comparable ranges in Mathematics, indicating robust item representation. All inter-factor co-variances were positive and significant, ranging from 0.201 to 0.695 in science and 0.115 to 0.558 in Mathematics, consistent with theoretical expectations. Reliability analyses (ω) indicated high internal consistency for all parcels (Science: 0.814–0.949; Mathematics: 0.785–0.946).

The factorial invariance of the five parcels was evaluated across the Science and Mathematics groups using a multiple-group CFA with parcels. As shown in Table 1, the configural model demonstrated a perfect fit (χ^2^ = 0.000, df = 0; CFI = 1.000, TLI = 1.000, RMSEA = 0.000, SRMR = 0.000), indicating that the hypothesized factor structure was equivalently represented across groups without imposing equality constraints. Subsequent testing of metric invariance, which constrained factor loadings to be equal across groups, yielded identical fit indices (χ^2^ = 0.000, df = 0; CFI = 1.000, TLI = 1.000, RMSEA = 0.000, SRMR = 0.000), suggesting that the magnitude of the relationships between observed parcels and their underlying latent factors was invariant across the two academic domains. Finally, scalar invariance, achieved by additionally constraining item intercepts, also maintained perfect fit (χ^2^ = 0.000, df = 0; CFI = 1.000, TLI = 1.000, RMSEA = 0.000, SRMR = 0.000), implying that both factor loadings and intercepts were equivalent across groups. Although Δχ^2^ statistics and associated *p*-values could not be computed due to estimation limitations with ordered parcels and sparse category frequencies, the standardized estimates and overall fit indices consistently indicate that the measurement model is fully invariant across the Science and Mathematics groups. These results provide strong psychometric support for the comparability of latent constructs and justify meaningful cross-group comparisons of factor means.

**Table 1:** Factorial invariance of the five parcels evaluated across the science and mathematics groups.

Model	χ^2^	df	p	CFI	TLI	RMSEA	SRMR	*Δχ* ^2^	Δdf	p (*Δχ*^2^)
Configural	0.000	0	NA	1.000	1.000	0.000	0.000	–	–	–
Metric	0.000	0	NA	1.000	1.000	0.000	0.000	NA	NA	NA
Scalar	0.000	0	NA	1.000	1.000	0.000	0.000	NA	NA	NA

#### 5.6.2 Descriptive Analysis of Teachers' Psychological Preparedness

The preliminary examination of teachers' psychological preparedness for AI-integrated teaching revealed nuanced patterns across cognitive, affective, and contextual dimensions. As summarized in Table 2, the mean scores suggest that participants exhibit moderate to high levels of readiness, motivation, and technophilia, reflecting a generally favorable orientation toward integrating AI into their pedagogical practice. Notably, the variable *Preparedness* (M = 94.59, SD = 13.52) demonstrates that teachers perceive themselves as substantially capable of incorporating AI tools within instructional contexts, highlighting an internalized confidence in their adaptive capacity. In contrast, the variables *Training Need* (M = 7.89, SD = 2.39) and *Tech Satisfaction* (M = 7.42, SD = 2.55) display higher variability, indicating that while teachers acknowledge the adequacy of existing technological resources, they remain consciously aware of gaps in formal professional development opportunities. These findings suggest a duality in psychological preparedness: an internal confidence in personal readiness coexisting with a recognition of external support needs. The near-normal distributions, as indicated by the skewness and kurtosis values, further validate the suitability of these data for subsequent network analyses, ensuring robust inference from multivariate exploratory methods.

**Table 2:** Descriptive statistics of psychological variables.

Variable	N	Mean	SD	Median	Skewness	Kurtosis
Preparedness	922	94.59	13.52	94	−0.268	0.346
Proficiency	922	4.91	2.58	5.00	0.19	−0.94
Training need	922	7.89	2.39	8.00	−0.88	−0.25
Tech satisfaction	922	7.42	2.55	8.00	−0.74	−0.40
Motivation	922	4.78	2.71	5.00	0.17	−0.88
Technophilia	922	5.28	2.92	5.00	0.20	−1.07

#### 5.6.3 Exploratory Graph Analysis (EGA)

**Figure 2:**
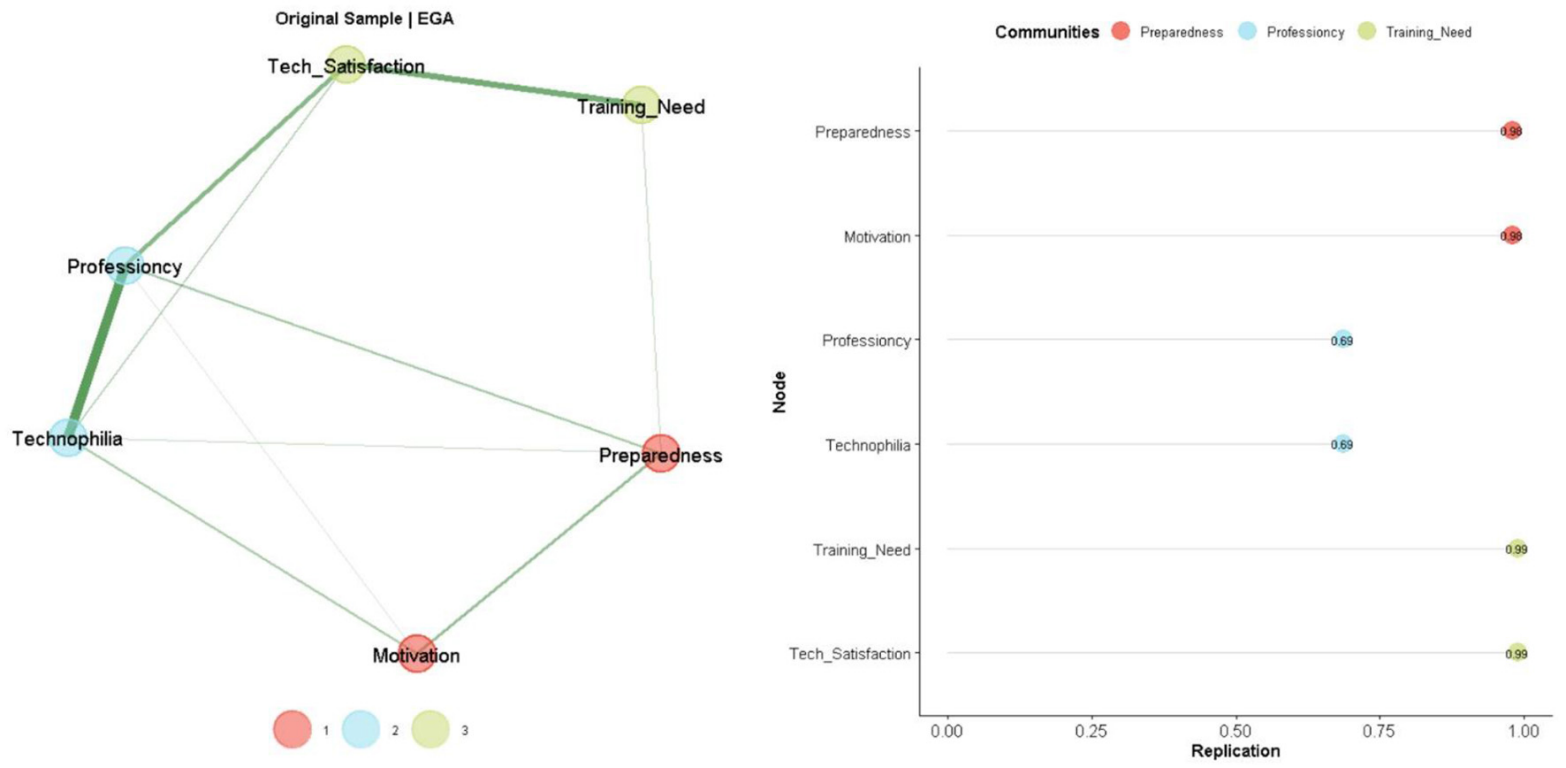
EGA variable clustering.

Applying Exploratory Graph Analysis (EGA) with the Glasso model and Walktrap community detection algorithm revealed a three-dimensional network structure, highlighting that psychological preparedness for AI integration is not a unitary construct but instead consists of interconnected, yet conceptually distinct, dimensions (see Figure 2). The network illustrates how personal, professional, and contextual factors coalesce to shape teachers' readiness for technological integration (Table 3).

**Table 3:** Bootstrap EGA results for dimensional stability.

Number of Dimensions	Frequency
1	0.6%
2	33.2%
3	66.2%

Dimension 1, comprising *Preparedness* and *Motivation*, reflects an internalized cognitive-affective orientation toward AI adoption. This dimension captures teachers' proactive engagement with pedagogical innovation, wherein intrinsic motivation aligns with self-perceived preparedness to implement AI tools effectively. Teachers who score highly on this dimension are likely to demonstrate not only the intention but also the psychological resilience to adopt and experiment with new instructional technologies. The interplay of motivation and readiness suggests that personal commitment is a primary driver of adaptive pedagogical behavior, reinforcing the notion that internal psychological states are foundational for successful AI integration.

Dimension 2, encompassing *Proficiency* and *Technophilia*, highlights the synergy between instructional expertise and a positive disposition toward technology. This dimension emphasizes that functional preparedness extends beyond mere motivation; effective AI integration requires both mastery of pedagogical principles and a favorable technological mindset. Teachers scoring high on this dimension can translate technological tools into instructional strategies, combining subject-matter expertise with enthusiasm for digital innovation.

Dimension 3, consisting of *Training Need* and *Tech Satisfaction*, represents an external or institutional layer of preparedness. This dimension reflects teachers' perceptions of resource adequacy, satisfaction with available technologies, and recognition of ongoing professional development needs. The co-occurrence of training awareness and satisfaction indicates that readiness is dynamically shaped by the broader educational ecosystem, emphasizing the importance of organizational support and resource availability in complementing individual motivation and competence.

The study's findings highlight several indicators that may constitute educational risks in the context of AI integration in teaching. First, the increasing need for teacher training reflects a gap between personal competencies and the knowledge required for effective use of digital tools, potentially leading to limited or inefficient classroom application. Second, fear of failure and technophobia emerge as psychological–cognitive barriers, reducing teachers' readiness to adopt innovative practices and increasing resistance to change. Finally, an imbalance between intrinsic motivation and institutional resources is evident, as insufficient infrastructure and organizational support can constrain the application of acquired skills, generating professional frustration and limiting the effective integration of emerging technologies. Although these indicators vary individually, they interact within a complex, interdependent system that underscores potential educational risks, emphasizing the necessity of integrated strategies encompassing continuous training, psychological support, and enhanced technological resources to strengthen teacher preparedness and foster a more adaptive and effective learning environment.

#### 5.6.4 Stability of dimensions using bootstrap EGA

The robustness of the three-dimensional solution was assessed using 500 bootstrap samples, yielding high stability and reproducibility. As shown in Table 4, the three-dimensional configuration emerged most frequently (66.2%), with a median number of dimensions of three and a 95% confidence interval spanning 2.04 to 3.96. These results affirm that the identified dimensions are not artifacts of sampling variability but represent consistently emergent psychological constructs across repeated resampling.

**Table 4:** Variables and their EGA-derived dimensions.

Variable	Dimension
Preparedness	1
Motivation	1
Proficiency	2
Technophilia	2
Training need	3
Tech satisfaction	3

The dimensional stability indices derived from bootstrap Exploratory Graph Analysis (EGA) primarily reflect the statistical reliability and robustness of the identified three-factor structure underlying teachers' psychological preparedness for AI integration. These indices indicate the consistency with which the latent dimensions, cognitive-affective readiness, professional-technological competence, and external resource orientation, emerge across resampled datasets, providing confidence in the measurement model. Importantly, stability itself does not constitute a pedagogical risk; rather, potential educational vulnerabilities are inferred from the levels and patterns of the constituent variables within these stable dimensions. For instance, consistently low scores in preparedness or high levels of technophobia within the robust factor structure could signify areas where teachers may struggle to adopt AI-enhanced instructional practices, thereby highlighting actionable targets for professional development and institutional support. Thus, while dimensional stability assures the reliability of the constructs, the identification of actual pedagogical risk depends on the content and directionality of the observed psychological indicators.

#### 5.6.5 Bivariate correlations among study variables

Prior to conducting the Exploratory Graph Analysis, bivariate correlations were computed to examine the pairwise relationships among the study variables and to test Hypothesis 3, which proposed that Technophilia and Training Need would show statistically significant associations with Instructional Proficiency. As presented in Table 5, Technophilia demonstrated a moderate positive correlation with Instructional Proficiency (*r* = 0.34, *p* < 0.001, 95% CI [.28,0.40]), indicating that teachers with greater enthusiasm for technology tend to perceive themselves as more proficient in AI-integrated instruction. Training Need showed a weak but statistically significant negative correlation with Instructional Proficiency (*r* = −0.12, *p* < 0.01, 95% CI−0.18,−0.06]), suggesting that higher perceived training needs are associated with slightly lower levels of self-reported proficiency. These findings provide initial support for Hypothesis 3 and confirm the interconnectedness of these constructs prior to network analysis. The magnitude and direction of these correlations align with theoretical expectations, supporting the construct validity of the single-item measures employed.

**Table 5:** Bivariate correlations with 95% confidence intervals.

Variable	1	2	3	4	5	6
1. Preparedness	-					
2. Proficiency	0.42^***^	-				
3. Motivation	0.38^***^	0.45^***^	-			
4. Technophilia	0.29^***^	0.34^*^ [.28,0.40]^**^	0.31^***^	-		
5. Training need	−0.08^*^	−0.12 [−0.18,−0.06]^**^	−0.06	0.03	-	
6. Tech satisfaction	0.15^***^	0.11^**^	0.09^*^	0.18^***^	0.22^***^	-

^*^p < 0.05,

^**^p < 0.01, ^**^p <0.001. Values in brackets represent 95% confidence intervals.

The bivariate correlation analysis provided initial support for Hypothesis 3, revealing that Technophilia was positively associated with Instructional Proficiency (*r* = 0.34, *p* < 0.001), while Training Need showed a modest negative association (*r* = −0.12, *p* < 0.01). These findings are particularly noteworthy given that all three variables were measured using single items, which, despite their efficiency in large-scale surveys, might be expected to attenuate observed correlations due to lower reliability (Allen et al., 2022). The fact that theoretically meaningful relationships emerged despite this potential attenuation underscores the robustness of the associations and supports the validity of the single-item measures employed.

However, the Exploratory Graph Analysis extended these bivariate findings by revealing that these variables do not operate as isolated pairwise relationships but instead form coherent psychological dimensions. Specifically, Proficiency and Technophilia clustered together in Dimension 2, suggesting that instructional expertise and technological disposition are intrinsically linked within teachers' readiness profiles. This clustering indicates that the correlation between these two variables reflects a deeper latent structure wherein they share common variance as components of a broader “professional-technological competence” dimension. Similarly, Training Need is grouped with Technology Satisfaction in Dimension 3, highlighting the contextual or institutional layer of preparedness.

Thus, while simple correlations capture the existence and direction of pairwise relationships among single-item measures, network analysis uncovers the latent structure organizing these relationships into broader, theoretically meaningful dimensions. This multilevel approach, examining both bivariate associations and network structures, provides a comprehensive understanding of how single-item indicators coalesce into stable psychological dimensions, addressing both the specificity of individual constructs and the emergent properties of the readiness network.

## 6 Discussion

The present findings highlight the central role of teachers' psychological readiness in shaping the effectiveness of AI-integrated teaching, particularly in science and mathematics education. The three-dimensional structure of preparedness identified in this study, comprising internal motivation and self-perceived preparedness, professional expertise coupled with technophilia, and external support factors, highlights the multifaceted nature of readiness. Consistent with prior research, teachers' attitudes toward technology, self-efficacy, and intrinsic motivation emerged as critical determinants of instructional choices and classroom practices (Nordlöf et al., 2019). Educators with high psychological readiness are more likely to engage proactively with AI tools, adopting innovative strategies and designing adaptive learning experiences tailored to diverse student needs (Dursun, 2019). Teachers who experience apprehension or lack confidence tend to rely on traditional, teacher-centered methods, which may constrain the potential of AI to enhance learning outcomes (Stumbriene et al., 2024; Joo et al., 2018).

These results further indicate that psychological readiness has a direct impact on student engagement and motivation. Teachers demonstrating confidence and enthusiasm in AI-enabled tools foster curiosity, sustained attention, and active participation in learning activities (Dursun, 2019; Joo et al., 2018). The integration of adaptive feedback mechanisms and personalized learning pathways allows psychologically prepared teachers to accommodate individual learning trajectories while promoting collaborative problem-solving and peer interaction (Nordlöf et al., 2019). Thus, psychological readiness extends beyond individual competence, influencing the broader social and emotional climate of the classroom. Internal motivational factors, when aligned with professional expertise, provide a foundation for pedagogical innovation and flexible teaching approaches.

At the classroom level, psychologically prepared teachers contribute to cultivating an open and progressive learning culture characterized by experimentation, critical inquiry, and higher-order thinking (Butler et al., 2024). Such teachers are more likely to implement AI-driven strategies that enable students to explore complex problems and develop competencies relevant to an AI-mediated society. Conversely, resistance or reluctance to adopt AI may result in rigid, less adaptive learning environments, limiting opportunities for student-centered practices and innovation (Butler et al., 2024). These findings emphasize that personal motivation, professional competence, and institutional support collectively determine both the quality of AI integration and the degree to which classrooms foster cognitive, social, and emotional engagement.

Equity and inclusivity are also intimately linked with teachers' psychological readiness. Well-prepared and confident teachers are better positioned to ensure that all students, regardless of their backgrounds or learning differences, benefit from AI-enhanced learning, including equitable access to resources, differentiated instruction, and careful monitoring of potential algorithmic biases (Jacobson, 2025; Joo et al., 2018). The institutional dimension of readiness, reflected in satisfaction with technological infrastructure and awareness of professional development needs, interacts with individual motivation to create conditions that support inclusive and effective teaching. Without sufficient support, even highly motivated teachers may encounter barriers to implementing AI equitably, highlighting the importance of holistic approaches that combine personal, professional, and contextual interventions.

Enhancing psychological readiness, therefore, requires strategies that address attitudes toward technology, self-efficacy, motivation, and awareness of training needs. Targeted professional development, ongoing mentoring, and institutional support structures that foster trust, ethical awareness, and shared responsibility are essential for sustaining engagement with AI in teaching (Jacobson, 2025; George Woodin, 2023). By cultivating these competencies, schools can establish innovative, adaptive, and inclusive learning environments that not only facilitate AI adoption but also prepare students to thrive in increasingly technology-driven educational contexts. Overall, the present findings reinforce the notion that psychological readiness is a multidimensional and dynamic construct, with far-reaching implications for both teacher practice and student outcomes in AI-integrated classrooms.

From a theoretical perspective, these findings resonate with constructivist principles; this readiness aligns closely with the principles of active knowledge construction and socially situated learning (Low et al., 2025; Lajoie and Li, 2023). AI-enabled systems, including adaptive learning platforms and real-time analytics, facilitate personalized instruction and differentiated feedback, allowing teachers to shift from knowledge transmitters to facilitators of learning. In science and mathematics classrooms, this constructivist alignment alleviates routine instructional burdens and enables teachers to focus on higher-order conceptual guidance, enhancing both perceived and actual teaching effectiveness (Vorsah and Oppong, 2024; Lajoie and Li, 2023). Teachers who perceive themselves as well-prepared are thus better equipped to leverage AI in ways that support active, student-centered learning.

Social learning theory further illuminates the role of psychological readiness in AI adoption by emphasizing observation, modeling, and interaction (Bandura, 1977; Strielkowski et al., 2025). AI-powered collaboration platforms, virtual laboratories, and simulation environments expand opportunities for social learning, allowing teachers to model expert reasoning, monitor collaborative inquiry, and guide peer interactions more effectively than in traditional settings (Gkintoni et al., 2025; Atabekova et al., 2025). Teachers with higher internal motivation and professional competence are more likely to engage with these tools, fostering adaptive instructional strategies and enhancing confidence in AI-supported practices. The interplay of competence, motivation, and technological disposition strengthens teachers' capacity to create socially connected, collaborative, and responsive learning environments.

Cognitive load theory (CLT) offers an additional explanatory lens by highlighting the mental effort required for complex learning tasks (Lajoie and Li, 2023; Gkintoni et al., 2025). AI systems reduce extraneous cognitive load by segmenting content, providing visual representations, and sequencing tasks adaptively, enabling teachers to focus on conceptual scaffolding and pedagogical decision-making. In this framework, teachers' preparedness and technophilia become critical, as confidence and openness to AI allow for more effective integration of cognitive support tools, while perceived training needs indicate areas where professional development could further enhance efficiency and instructional quality (George and Wooden, 2023; Atabekova et al., 2025). This alignment suggests that teachers' psychological readiness directly interacts with AI's capacity to scaffold complex learning, supporting both student comprehension and teacher efficacy.

The concept of interconnected learning further contextualizes the findings by emphasizing the formation of networks across digital tools, human actors, and information systems (Gkintoni et al., 2025; Sajja et al., 2024). Teachers' effectiveness in AI-integrated classrooms depends on their ability to orchestrate these networks, coordinating learning across technological and social resources. High levels of professional motivation and pedagogical competence enable teachers to interpret learning analytics, adapt instructional strategies, and sustain adaptive learning environments. Technophilia plays a pivotal linking role, connecting motivation, competence, and training needs to create dynamic, responsive readiness profiles (Hu et al., 2025; Sun et al., 2023). Teachers with strong technological inclination are more likely to engage in targeted professional development, sustain motivation during the integration process, and apply AI tools thoughtfully to enhance learning outcomes.

Professional development emerges as a critical external factor supporting psychological readiness. AI integration in science and mathematics classrooms requires teachers to develop not only technical proficiency but also pedagogical judgment, reflective practice, and ethical awareness (Acosta-Enriquez et al., 2025; Hu et al., 2025). The findings indicate that perceived training needs interact dynamically with competence, motivation, and technophilia, shaping teachers' overall readiness for AI-supported instruction (Bakracheva and Mizova, 2024; Simut et al., 2024). Continuous, adaptive professional development, particularly when embedded within mentoring, peer collaboration, and learning communities, enhances teacher confidence, fosters sustained engagement, and facilitates the practical application of AI tools (Stumbriene et al., 2024; Howard et al., 2025). These results suggest that supporting psychological readiness through tailored professional learning is essential for fostering innovative, equitable, and effective AI-enhanced classrooms.

## 7 Conclusion

The present study demonstrates that teachers' psychological readiness for AI-integrated teaching is inherently multidimensional, as reflected in the three distinct communities of variables identified through Exploratory Graph Analysis (EGA). The first community, encompassing internal motivation and self-perceived preparedness, highlights the foundational role of cognitive-affective engagement in driving proactive adoption of AI tools. This cluster supports constructivist principles, where teachers act as facilitators of learning, designing adaptive and personalized instructional experiences that scaffold student knowledge construction (Low et al., 2025; Lajoie and Li, 2023). The second community, consisting of pedagogical competence and technophilia, underscores the interdependence between instructional expertise and positive technological disposition. This linkage aligns with social learning and interconnected learning theories, illustrating how teachers' mastery of content and enthusiasm for digital innovation enable effective orchestration of collaborative and technology-mediated learning networks (Bandura, 1977; Gkintoni et al., 2025; Sajja et al., 2024). The third community, capturing training needs and technology satisfaction, emphasizes the external or institutional dimension of readiness, highlighting the critical influence of professional development and resource adequacy on sustaining effective AI integration. This cluster resonates with cognitive load theory, as structured support reduces extraneous demands on teachers' cognitive resources, allowing for focused, evidence-based pedagogical decision-making (Lajoie and Li, 2023; George and Wooden, 2023). The EGA-derived communities not only corroborate the study's descriptive and inferential findings but also provide a theoretically coherent framework that links internal motivation, professional competence, technological inclination, and institutional support to enhanced teaching efficacy and adaptive classroom environments. These results underscore that effective AI integration is not solely a technological challenge but a complex interplay of psychological, pedagogical, and contextual factors, offering empirical and conceptual guidance for designing targeted professional development, fostering teacher confidence, and promoting inclusive, innovative learning practices in AI-enhanced education.

## 8 Limitations

While this study provides a robust, network-informed understanding of teachers' psychological preparedness for AI integration, several limitations must be acknowledged. First, the cross-sectional design precludes causal inference, limiting our ability to determine the directionality of relationships among motivational, competency, and contextual factors. Second, the reliance on self-reported measures, though practical, may introduce biases such as social desirability or common method variance, potentially affecting the accuracy of perceived competence and motivation. Third, the use of item parceling, while improving model estimation, may obscure nuanced item-level dynamics and mask potential multidimensionality within the latent constructs. Fourth, the sample, though sizable, was drawn from a specific cultural and institutional context (science and math teachers), which may limit the generalizability of the dimensional structure and network relationships to educators in other regions or educational systems. Finally, while factorial invariance was supported, the estimation faced challenges due to sparse category frequencies in ordinal data, warranting caution in interpreting strict scalar invariance. Future longitudinal and mixed-methods research across diverse contexts is recommended to validate these findings and explore the temporal evolution of the readiness network.

### 8.1 Theoretical and Practical Implications

The findings of this study provide significant theoretical and practical insights into teachers' psychological readiness for AI-integrated education. The three communities of variables identified internal motivation and self-perceived preparedness, pedagogical competence coupled with technophilia, and external support factors, which support a multidimensional and dynamic conceptualization of readiness. Theoretically, these clusters illustrate how cognitive, professional, and contextual elements interact to shape adaptive teaching practices. Internal motivation and self-efficacy highlight the role of teachers as facilitators of active, student-centered learning. Pedagogical competence combined with technophilia emphasizes how expertise and a positive disposition toward technology enable teachers to orchestrate collaborative and adaptive AI-supported learning environments. External support factors, including training needs and satisfaction with technological resources, underscore the importance of institutional scaffolding in promoting effective instructional decisions and reducing cognitive burdens.

Practically, the study emphasizes the necessity of holistic and sustained professional development initiatives. Effective interventions should address the interplay of technical skills, pedagogical competence, motivation, and confidence in applying AI tools. Mentoring, collaborative learning communities, and ongoing support are crucial for helping teachers translate technological affordances into meaningful instructional strategies. Moreover, psychological readiness plays a central role in promoting equity and inclusivity. Teachers who are confident and well-prepared are better positioned to provide differentiated instruction, ensure equitable access to AI resources, and monitor potential biases, supporting diverse learners and fostering inclusive classroom environments.

### 8.2 Future Directions

Future research should investigate the dynamic interplay of internal motivation, pedagogical competence, technophilia, and external support in shaping teachers' psychological readiness for AI-integrated education, ideally through longitudinal and multilevel designs that capture changes over time and across diverse educational contexts. Expanding the use of network-based approaches, such as Exploratory Graph Analysis, to integrate teacher, classroom, and student-level variables could clarify how readiness translates into instructional practices, student engagement, and learning outcomes. Additionally, experimental studies examining the effectiveness of targeted professional development, mentoring, and collaborative learning communities are needed to identify strategies that strengthen motivation, competence, and technological disposition. Attention to equity and inclusivity should remain central, exploring how teachers' readiness interacts with resource access, student diversity, and potential algorithmic biases to foster fair and effective learning experiences. By addressing these avenues, future work can advance both theoretical understanding and practical implementation of AI in education, ultimately supporting adaptive, innovative, and inclusive teaching practices.

## Data Availability

The original contributions presented in the study are included in the article/supplementary material, further inquiries can be directed to the corresponding author.
